# A comparative study on the persistence and viability of SARS-CoV-2 wild-type and omicron variant on artificially contaminated surfaces: the role of fomites

**DOI:** 10.1080/22221751.2023.2239941

**Published:** 2023-08-02

**Authors:** Giovanna Fusco, Gerardo Picazio, Lorena Cardillo, Alessia Pucciarelli, Luisa Marati, Federica Di Maggio, Marcella Nunziato, Sergio Brandi, Esterina De Carlo, Claudio de Martinis, Francesco Salvatore

**Affiliations:** a Department of Animal Health, Istituto Zooprofilattico Sperimentale del Mezzogiorno, Naples, Italy; b CEINGE-Biotecnologie Avanzate Franco Salvatore, Naples, Italy; c Department of Molecular Medicine and Medical Biotechnologies, University of Naples Federico II, Naples, Italy

**Keywords:** SARS-CoV-2 lineage B.1, BA.1 omicron variant, artificial contamination, virus stability, inanimate surfaces

## Abstract

Indirect transmission of Severe Acute Respiratory Syndrome Coronavirus 2 (SARS-CoV-2) has been investigated but it is still not completely understood. The present study aimed to compare the persistence and viability of the lineage B.1 and omicron BA.1 subvariant in five daily-use materials to evaluate the role of fomites as a possible source of infection. Artificial contamination was performed in the first set of materials, ethylene vinyl acetate (EVA), cardboard, polystyrene, aluminium, and plastic. Further surfaces using BA.1 (glass, plexiglass, cotton, polyester, and tetrapak) were conducted. The persistence, viability of Vero E6 cell cultures and the residual infectivity of the two lineages were evaluated over 5 days. The results showed different stabilities between the tested matrices. In cotton and polyester, the RNA was undetectable in 24 and 48h post-contamination (p.c.), respectively, and the virus was not viable within 30 min, while in the other surfaces, both lineages, RNA was detectable until 120h p.c. A rapid decay of the viral load was revealed on cardboard, mostly for the omicron variant. Furthermore, on all the materials, longer stability of BA.1 was demonstrated, but showing a less intense CPE than the wild-type. EVA was the material that was able to better sustain virus stability as the virus developed CPE up to 72h p.c. In conclusion, the potential spread of SARS-CoV-2 through fomites is conceivable, albeit it is difficult to establish the real capacity to infect people. Nevertheless, thise information is fundamental to adopting the appropriate measures to mitigate the spread of SARS-CoV-2 and its variants.

## Introduction

During the almost three-year pandemic, the occurrence of numerous mutations within the SARS-CoV-2 genome has led to the emergence of several variants, some of them showing enhanced morbidity, mortality or the ability to escape the host immune system. These variants are known as Variants of Concern (VOCs). The latest VOC, the omicron variant and its sub-variants, seems evolved separately from other circulating SARS-CoV-2 lineages [1] showing at least 37 mutations in the *Spike* protein, and 15 of them are localized within the Receptor Binding Domain (RBD), being responsible for the mechanism of immune-evasion, with the ability to escape from antibodies generated by previous infection, vaccination and antibody-based treatments [2,3]. Furthermore, it is characterized by higher transmissibility due to a three-fold higher binding affinity for the Angiotensin-converting enzyme 2 (ACE-2) [2,4] mostly for the upper respiratory tract than previous VOCs [1,5]. Indeed, increased viral loads in the oral swab of infected individuals, prolonged viral shedding as well as higher environmental stability have been observed [6], which might have played a key role in the sharp overtake of the previous circulating delta VOC [3,7]. All these features represent the basis for rapid diffusion in the case of respiratory viruses, which can spread mainly through direct and indirect contact via fomites [8,9,10]. Accordingly, contaminated surfaces might represent an important tool for viral spreading in particular settings, such as hospitals, nursing facilities, schools and kindergartens, restaurants and gyms [11–15].

SARS-CoV-2 can persist on inanimate surfaces from days to weeks [16–19]; nevertheless, this aspect does not necessarily imply that surfaces can certainly operate as fomites. Indeed, SARS-CoV-2 indirect transmission through fomites has been hypothesized, but there is still a lack of conclusive proof and a few cases suggest the role of contaminated surfaces in the epidemiology of the virus [13,20].

The identification of SARS-CoV-2 RNA by reverse-transcription PCR (RT–PCR) has been frequently used to determine the contamination of the surfaces [21,22]; however, this method does not always reflect the real infectivity of the surface, so that studies on the viability of the virus are also needed to have conclusive responses.

To better understand the effectiveness of indirect transmission through fomites, the current study attempted to investigate the viability of SARS-CoV-2 BA.1 omicron subvariant on artificially contaminated surfaces and the viral load kinetics over time, also obtaining comparative results with the lineage B.1.

## Materials and methods

### Artificial contamination of the surfaces

**Figure 1.**
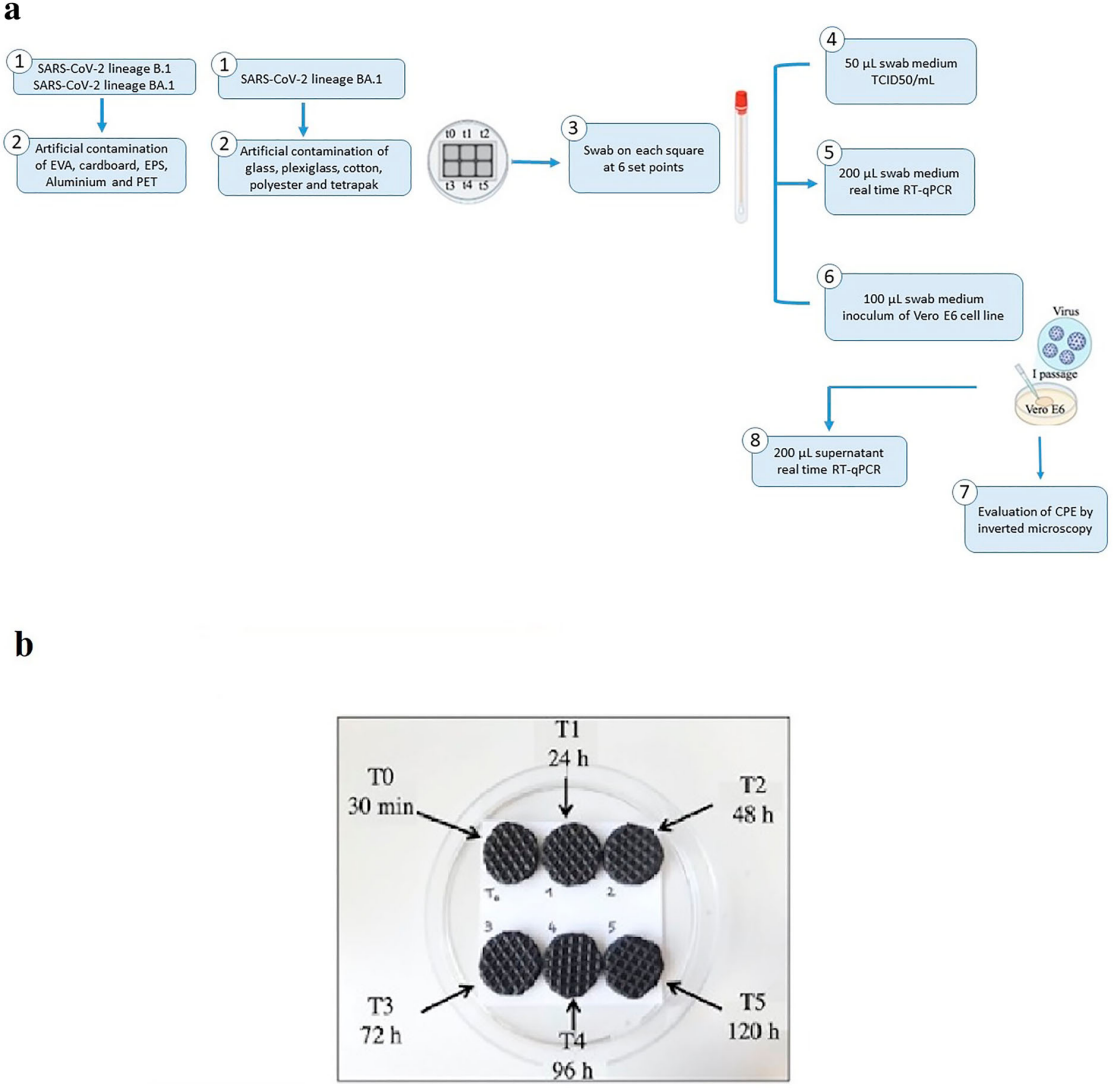
**(a)** Representative flowchart of all the steps of the experiments. SARS-CoV-2 B1 and BA.1 lineage were used to contaminate five different materials, Ethylene vinyl acetate (EVA), Cardboard, Expanded Polystyrene (EPS), Aluminium, and Polyethylene Terephthalate (PET**)**. Furthermore, five additional surfaces were artificially contaminated using only the BA.1 lineage. Thereby, at six different set points, starting from T0, corresponding to 30 min post-contamination and then every 24 h up to 120 h, a swab was administrated to the relative circle (**b**) and suspended in 1 mL Universal Transport Medium (UTM). Next, aliquots of 50 µL of UTM were collected to perform TCID50/mL, 100 µL was used as inoculum for Vero E6 cells and 200 µL to carry out real-time RT-qPCR to evaluate viral loads. Subsequently, cell cultures were inspected for the evaluation of the development of cytopathic effect (CPE) by inverted microscopy. When at least 50% CPE occurred or, after 7 days post-cell infection, the supernatant was collected and viral loads were evaluated. (**b)** An example of the scheme of artificial contamination of Ethylene vinyl acetate (EVA). Each circle represents a specific time for sample collection. Time 0 (T0): 30 min post-contamination; time 1 (T1): 24 h; time 2 (T2): 48 h, time 3 (T3): 72 h; time 4 (T4): 96 h and time 5 (T5): 120 h.

The artificial contamination of five different materials, ethylene vinyl acetate (EVA), cardboard, expanded polystyrene (EPS), aluminium and polyethylene terephthalate (PET), was carried out using SARS-CoV-2 lineage B.1 hCoV-19/Italy/CAM-INMI-32803-66/2020 and the BA.1 omicron subvariant hCoV-19/Italy/CAM_IZSM_RD20701948219_IZSM_COLLI_TIGEM/2022, which were titrated using the Endpoint Dilution Assay (TCID50/mL) according to the Spearman–Kärber method [23]. A solution containing 10^5^ TCID50/mL in 50 µL for both viruses was obtained, corresponding to 1.5E+07 copies/µL and 1,35E+07 copies/µL. Moreover, the omicron variant was also used to perform the artificial contamination of five additional materials, glass, plexiglass, cotton, polyester, and tetrapak. The whole procedure is schematized in Figure 1(a).

The surfaces were previously checked for the absence of SARS-CoV-2 by real-time RT–PCR (as reported below) and sterilized by UV radiation, as described by Heilingloh and colleagues [24].

Each material was cut into 6 equal surfaces of 0.8 cm in diameter of 10^5^ TCID50 in 50 µL viral solution and was dispensed onto each area. Next, the prepared surfaces were placed into petri dishes, covered and incubated at 22.5°C and relative humidity (RH) of 58.6% for subsequent analyses. For all the examined surfaces, each circle represented a specific time post-contamination, as shown in Figure 1(b). Furthermore, in separate petri dishes, cytotoxicity controls were included for all the tested surfaces by placing 50 µL medium without any virus on each surface that underwent the same procedures in parallel with the contaminated ones.

Thus, starting from 30 min p.c., indicated as T0, samples were collected every 24 h up to 120 h p.c., identified from T1 to T5 (Figure 1(b)), by rubbing the infected surface [17] through transverse, horizontal, and vertical movements, using sterile nylon swabs in the corresponding area of the contaminated sections and placed into the Universal Transport Medium (UTM) (Copan, Brescia, Italy). Aliquots of 200 µL of UTM were collected from each swab for molecular analysis, another 100 µL was used as cell inoculum, to evaluate RNA persistence and viability of the two lineages, respectively and a final aliquot of 50 µL was collected to carry out TCID50/mL (reported below) (Figure 1(a)) to titrate the virus on the surfaces. The supernatant of the primary cell infection was then used to verify the viral loads by RT-qPCR and the viability of the virus through the development of CPE.

### Virological analyses

#### Cell cultures

Vero E6 cells (ATCC Vero C1008) were provided by the OIE Collaborating Centre for Veterinary Biological Biobank (Istituto Zooprofilattico Sperimentale della Lombardia e dell’Emilia Romagna, IZSLER, Brescia, Italy) at passage 35 with the certification of the absence of *Mycoplasma spp* and viruses. Before the infection, Vero E6 cells were cultured as described by the European Collection of Authenticated Cell Cultures (ECACC) and 24-well plates were prepared by seeding approximately 150,000 cells/well in Eagle Minimum Essential Medium (EMEM) (Gibco, Life Technologies, Europe B.V. Bleiswijk, Netherlands) integrated with 10% foetal bovine serum (FBS) (Gibco, Life Technologies, Europe B.V. Bleiswijk, Netherlands), 1% antibiotic-antimycotic (Gibco, Life Technologies, Europe B.V. Bleiswijk, Netherlands), 2 mM L-glutamine (Gibco, Life Technologies, Europe B.V. Bleiswijk, Netherlands), incubated at 37 ± 1°C in 5% CO_2_ atmosphere until 80% cell confluence was reached. Next, 1 mL of UTM was filtered through a 0.45 µm pore size syringe filter (Millipore, Thermo Fisher Scientific, Waltham, MA, USA) and 100 µL was used as cell inoculum, incubated for 30 min at 37°C and, finally, 1 mL complete EMEM with 2% FBS, was added in each well. All the experiments were conducted in the presence of a negative control, using EMEM medium as inoculum, and a positive control. Next, an inverted microscope at 5X magnification (Axiovert 25 inverted microscope, Carl Zeiss, Oberkochen, Germany) using AxioVision 4.8 software, was used to inspect the monolayers after 24 h for the visualization of the cytotoxic effect and up to 7 days for the development of the cytopathic effect (CPE).

Next, quantification of the infectious particles for the residual infectivity of the virus on the surfaces was carried out using TCID50 assay on Vero E6 cells. In brief, 50 µL of UTM was collected from the swab and aliquots of 10 µL were used in duplicates to infect 80% confluent Vero E6 cells on 96-well plates with 10-fold serially diluted virus to reach 10^−6^ final dilution. The infected cells were incubated at 37° C with 5% CO2 and daily inspected from 2 up to 7 days post-infection to evaluate the development of CPE by cristal violet staining [23,25].

#### Molecular analyses

For the assessment of the viral loads, biomolecular examinations were conducted using real-time RT-qPCR. Aliquots of 200 µL were collected from UTM, cell culture and sub-culture cryo-lysates with the addition of 200 µL TRIzol (Ambion, Life Technologies, Carlsbad, CA, US) to perform nucleic acid extraction and purification with KingFisher Flex (Thermo Fisher Scientific, Waltham, MA, USA), using the MVP_2Wash_200_Flex programme, following the manufacturer’s instructions. The elution was carried out in a 50-µL final volume and stored at –80°C until use. RT-qPCR analysis was conducted using TaqPath COVID-19 CE-IVD RT–PCR kit (Thermo Fisher Scientific, Waltham, MA, USA). Amplification settings, thermal profile, and viral loads were performed according to the protocol already described by Cardillo and colleagues [26].

The association of the development of CPE and the increase in viral loads over time by RT-qPCR allowed us to assess the stability and viability of the virus, which was titrated using the TCID50/mL assay.

## Results

In the first set of experiments, a comparison between SARS-CoV-2 lineage B.1 and BA.1 omicron subvariant persistence and stability was conducted on a set of five materials composed of four non-porous surfaces, Ethylene Vinyl Acetate (EVA), expanded polystyrene (EPS), aluminium and Polyethylene Terephthalate (PET), and cardboard as a porous material.

On these tested surfaces the RNA was detectable during the whole experiment (120 h post contamination) for both the lineages (Table 1); nevertheless, rapid decay of viral loads was observed in cardboard, more markedly for the BA.1 variant, which showed a 3 log reduction of the concentration within 24 h, thereby remaining almost stable from 4.34E+01 (T1) to 1.39E+01 (T5). Furthermore, when inoculated onto the cells, a CPE was observed at 30 min p.c., with a very low virus titre, while soon after it was inactivated. Thus, from 24 to 120 h p.c., no viral target and no CPE were detectable.

**Table 1.** The results of the comparison of lineage B.1 and BA.1 viral loads and viability in the first set of materials.

Ethylene Vinyl Acetate (EVA)										
	Lineage B.1 viral load (copies/µL)					Lineage BA.1 viral load (copies/µL)				
Time (h)	Swab mean	Swab SD	TCID50/mL	Cell passage mean	Cell passage SD	Swab mean	Swab SD	TCID50/mL	Cell passage mean	Cell passage SD
0.5	6,79E+05	3,73E+05	1,00E+03	2,28E+06	1,59E+06	3,09E+05	1,22E+05	1,00E+03	5,30E+06	2,35E+06
24	3,78E+05	1,26E+05	1,00E+01	1,66E+06	5,94E+05	2,87E+05	8,33E+04	4,64E+01	5,46E+06	3,75E+06
48	1,08E+05	2,79E+04	-	2,58E+04	7,57E+03	2,91E+05	1,81E+05	1,00E+01	2,30E+06	1,90E+06
72	1,35E+05	9,57E+04	-	3,80E+02	2,55E+02	2,44E+05	5,30E+04	-	1,59E+06	2,09E+06
96	1,62E+04	1,25E+04	-	5,38E+02	3,36E+02	3,02E+05	1,26E+04	-	1,81E+04	2,69E+03
120	1,42E+04	1,86E+04	-	4,73E+02	5,27E+02	1,42E+05	8,39E+04	-	7,87E+03	1,23E+03
Time (h)	Cardboard									
0.5	4,51E+05	2,56E+05	3,16E+01	2,30E+06	7,07E+05	6,17E+04	4,57E+04	3,16E+01	5,87E+06	3,43E+06
24	6,98E+03	2,06E+03	-	2,56E+04	3,60E+04	4,34E+01	1,53E+01	-	0,00E+00	0,00E+00
48	2,11E+03	2,18E+03	-	2,58E+04	3,29E+04	4,00E+01	2,64E+01	-	0,00E+00	0,00E+00
72	2,05E+03	2,34E+03	-	7,63E+03	6,46E+03	4,45E+01	2,33E+01	-	0,00E+00	0,00E+00
96	3,51E+03	5,71E+03	-	4,36E+02	2,70E+02	1,63E+01	5,51E+00	-	0,00E+00	0,00E+00
120	2,82E+03	3,77E+03	-	6,00E+01	5,66E+01	1,39E+01	2,61E+00	-	0,00E+00	0,00E+00
Time (h)	Aluminium									
0.5	4,13E+05	2,56E+05	1,00E+03	2,74E+06	1,50E+06	4,33E+05	2,24E+05	2,15E+03	3,00E+06	2,09E+06
24	4,62E+05	1,98E+05	3,16E+02	2,23E+06	9,97E+05	3,58E+05	8,86E+04	6,81E+02	2,45E+06	2,05E+06
48	8,57E+04	2,21E+04	–	1,88E+03	6,01E+02	2,76E+05	2,21E+05	1,00E+01	1,35E+06	3,75E+05
72	7,83E+04	1,05E+05	–	1,40E+03	1,48E+02	2,38E+05	3,74E+05	-	1,76E+04	6,36E+02
96	8,61E+04	5,95E+04	–	6,16E+02	1,90E+02	1,97E+05	2,52E+05	-	1,47E+04	2,33E+03
120	1,95E+04	3,83E+03	–	2,83E+02	1,06E+01	1,12E+05	5,29E+04	-	9,45E+03	7,00E+03
Time (h)	Expanded Polystyrene (EPS)									
0.5	4,67E+05	2,96E+05	3,16E+01	3,55E+06	4,95E+05	3,60E+05	1,06E+05	6,81E+03	3,82E+06	2,96E+06
24	4,65E+05	2,89E+05	1,00E+01	2,45E+06	1,06E+06	2,86E+05	4,90E+04	6,81E+02	1,20E+06	4,48E+05
48	1,26E+05	6,04E+04	–	2,81E+03	1,69E+03	2,93E+05	2,11E+05	3.16E+01	4,05E+06	5,61E+06
72	1,72E+05	1,27E+05	–	1,48E+03	1,64E+03	2,71E+05	4,42E+04	-	1,50E+04	8,34E+03
96	1,33E+04	6,43E+02	–	3,91E+02	8,63E+01	2,97E+05	1,20E+05	-	1,14E+04	7,33E+03
120	4,36E+03	5,66E+02	–	1,75E+02	3,61E+01	2,84E+05	1,81E+05	-	6,31E+03	4,68E+03
Time (h)	Polyethylene Terephthalate (PET)									
0.5	5,36E+05	2,75E+05	1,46E+04	2,06E+06	3,68E+05	3,92E+05	1,83E+05	3,16E+03	1,69E+06	3,32E+05
24	3,07E+05	2,21E+05	–	1,06E+05	1,33E+05	2,95E+05	1,05E+05	4,64E+02	1,59E+06	7,07E+04
48	1,44E+05	6,21E+04	–	2,73E+04	3,77E+04	2,76E+05	2,32E+05	-	1,31E+03	5,80E+01
72	1,09E+04	8,41E+03	–	2,60E+04	3,43E+04	3,85E+04	2,90E+04	-	3,48E+03	3,50E+03
96	1,14E+04	1,72E+04	–	1,74E+04	2,31E+04	3,12E+03	4,05E+03	-	4,49E+03	5,21E+03
120	1,01E+04	1,28E+04	–	5,65E+02	1,91E+02	1,29E+03	1,48E+03	-	6,41E+03	8,75E+03

**Figure 2.**
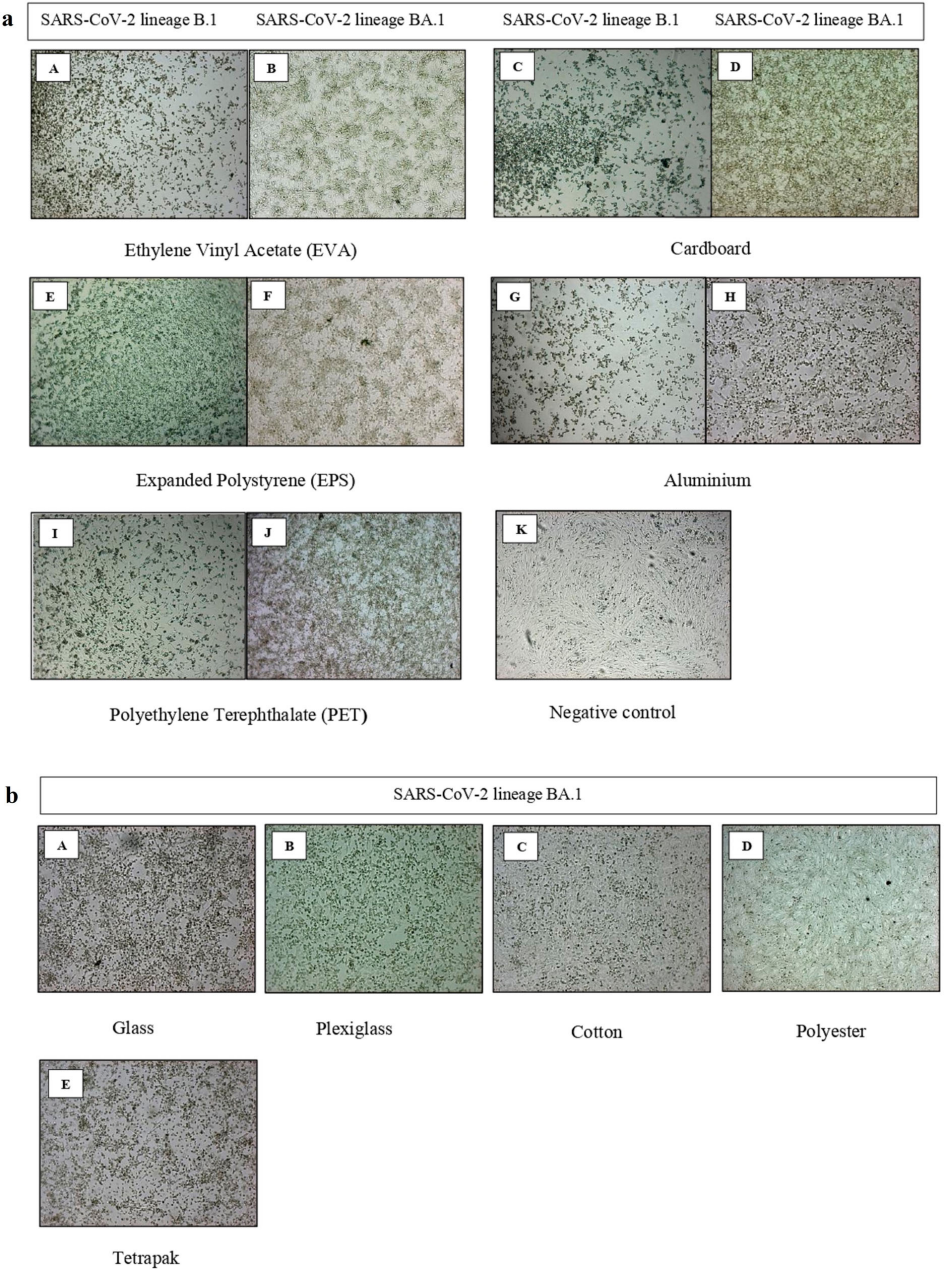
**(a)** Comparison of the cytopathic effects (CPEs) of SARS-CoV-2 lineage B.1 and BA.1 in the first set of materials. (**A**) Cytopathic effects induced by SARS-CoV-2 lineage B.1 on Vero E6-infected cells obtained from Ethylene Vinyl Acetate collection at T1 sample time (24 h) for B.1 and at T4 (72 h) for the omicron variant (**B**). Cytopathic effects induced by SARS-CoV-2 lineage B.1 (**C**) and BA.1 (**D**) on Vero E6 infected cells obtained from Cardboard collection at T0 sample time (30 min). (**E**) Cytopathic effects induced by SARS-CoV-2 lineage B.1 on Vero E6-infected cells obtained from Polystyrene collection at T1 sample time (24 h) for B.1 and at T2 (48 h) for the omicron variant (**F**). **(G**) Cytopathic effects induced by SARS-CoV-2 lineage B.1 on Vero E6-infected cells obtained from aluminium collection at T1 sample time (24 h) for B.1 and at T2 (48 h) for the omicron variant (**H**). **(I**) Cytopathic effects induced by SARS-CoV-2 lineage B.1 on Vero E6-infected cells obtained from Polyethylene Terephthalate collection at T0 sample time (30 min) for B.1 and at T1 (24 h) for the omicron variant (**J**). (**K**) Negative control at T5 (120 h post inoculum) showing a completey intact cell monolayer. **(b)** Cytopathic effect induced by SARS-CoV-2 lineage BA.1 omicron variant for the second set of materials. Cytopathic effects on Vero E6-infected cells obtained from glass (**A**) and plexiglass (**B**) at T2 (48 h) sample time. No visible CPE in cotton (**C**) and polyester (**D**) at T0 (30 min) sample time. Cytopathic effects on Vero E6-infected cells obtained from tetrapak (**E**) at T1 (24 h) sample time. Microscopy images by inverted microscope at 5× magnification.

**Swab**: viral load performed directly on the surfaces, starting from 30 min post-artificial contamination, indicated as 0.5 h (T0), and every 24 h up to 120 h post-contamination (T1–T5). **Cell passage**: viral load performed on cell supernatant after three cycles of frosting and towing. All the procedures were carried out in triplicates, thus results are reported as **mean** values and **Standard Deviation (SD).** The association of the increase in viral loads between swabs and cell passages (in bold) and the presence of a cytopathic effect (CPE) on the cells (Figure 2), was used to assess the viability of the virus, followed by **TCID50/mL** assay to evaluate the titre of viable virus on the surfaces.

On non-porous surfaces, it was possible to notice a clear difference in the stability and viability of the two lineages, as the omicron variant showed at least 24 h longer-lasting residual infectivity than the lineage B.1, albeit the intensity of the CPE was less intense (Figure 2(a)).

The highest stability was observed on EVA, where it was shown an increase in viral loads between the surface and the cell passage and the development of the CPE that lasted up to 24 h post-cell inoculum for the lineage B.1, compared to the 72 h of the omicron variant. Despite this, a virus titre was obtained at a maximum of 48 h p.c. showing a very low amount of viable virus.

The different behaviour of SARS-CoV-2 on the basis of material features was also demonstrated in the second set of materials when the omicron variant was used to artificially contaminate glass, plexiglass, cotton, polyester, and tetrapak (Table 2).

**Table 2.** Results of BA.1 Omicron variant persistence and viability on the second set of materials.

Lineage BA.1 viral load (copies/µL)										
Time (h)	Glass					Plexiglass				
	Swab mean	Swab SD	TCID50/mL	Cell passage mean	Cell passage SD	Swab mean	Swab SD	TCID50/mL	Cell passage mean	Cell passage SD
0.5	1,67E+05	1,26E+05	6,81E+03	2,30E+06	4,24E+05	2,40E+05	1,21E+05	1,00E+03	1,14E+06	3,10E+05
24	1,55E+05	1,05E+05	6,81E+02	1,11E+06	4,13E+05	1,51E+05	1,06E+05	1,00E+02	1,06E+06	1,98E+05
48	1,20E+05	8,42E+04	1,00E+01	5,35E+05	6,01E+05	1,55E+05	1,21E+05	1,00E+01	5,83E+05	5,34E+05
72	4,12E+04	6,88E+03	–	2,98E+03	3,11E+02	1,02E+05	1,51E+04	–	1,93E+05	9,76E+04
96	5,09E+03	3,44E+02	–	9,81E+02	1,16E+03	1,07E+05	1,15E+04	–	5,34E+03	9,05E+02
120	9,27E+02	1,25E+02	–	1,36E+02	9,05E+01	7,99E+04	9,85E+03	–	4,01E+03	3,83E+03
Time (h)	Cotton					Polyester				
0.5	3,57E+02	4,53E+02	–	0,00E+00	0,00E+00	7,30E+01	2,34E+01	–	0,00E+00	0,00E+00
24	9,87E+01	1,40E+02	–	0,00E+00	0,00E+00	0,00E+00	0,00E+00	–	0,00E+00	0,00E+00
48	0,00E+00	0,00E+00	–	0,00E+00	0,00E+00	0,00E+00	0,00E+00	–	0,00E+00	0,00E+00
72	0,00E+00	0,00E+00	–	0,00E+00	0,00E+00	0,00E+00	0,00E+00	–	0,00E+00	0,00E+00
96	0,00E+00	0,00E+00	–	0,00E+00	0,00E+00	0,00E+00	0,00E+00	–	0,00E+00	0,00E+00
120	0,00E+00	0,00E+00	–	0,00E+00	0,00E+00	0,00E+00	0,00E+00	–	0,00E+00	0,00E+00
Time (h)	Tetrapak									
0.5	1,56E+05	1,52E+05	2,15E+03	1,06E+06	1,21E+06					
24	1,45E+05	1,23E+05	3,16E+02	8,93E+05	1,00E+06					
48	1,08E+05	2,02E+04	–	2,51E+03	2,26E+02					
72	4,63E+04	3,88E+04	–	5,39E+03	5,90E+03					
96	2,80E+04	1,70E+03	–	2,09E+03	3,04E+02					
120	2,98E+04	9,65E+03	–	9,94E+02	8,49E+00					

**Swab**: viral load performed directly on the surfaces, starting from 30 min post-artificial contamination, indicated as 0.5 h (T0), and every 24 h up to 120 h post-contamination (T1-T5). **Cell passage**: viral load performed on cell supernatant after three cycles of frosting and towing. All the procedures were carried out in triplicates, thus results are reported as **mean** values and **Standard Deviation (SD).** The association of the increase in viral loads between swabs and cell passages (in bold) and the presence of a cytopathic effect (CPE) on the cells (Figure 2), was used to assess the viability of the virus, followed by **TCID50/mL** assay to evaluate the titre of viable virus on the surfaces.

In polyester and cotton, the virus was rapidly inactivated, thus showing a small amount of recovered RNA on the surfaces as soon as 30 min and 24 h post contamination, respectively. This aspect was reflected in the cells, where it was observed a completely intact monolayer and no recovered RNA since T0. On the other hand, in the other three non-porous smooth materials, glass, plexiglass, and tetrapak, the virus was viable for up to 48 h (Figure 2(b)).

## Discussions

Respiratory viruses, including SARS-CoV-2, are disseminated through aerosolization of droplets containing the virus, so that the deposition of infected droplets on inanimate surfaces may lead them to act as a potential source of infection.

Despite the transmission of SARS-CoV-2 via inanimate objects has been raised in the scientific community as soon as the virus emerged at the end of 2019 [27], currently, there is no complete evidence supporting the link between SARS-CoV-2 infection and the role of fomites [28,29]. Limited information is available for several surfaces that come into contact with people in everyday life. EVA, for instance, is a flexible, stable and inexpensive material that is often employed to realize tools and surfaces, such as play and gym mats, often in contact with children and adults during gym and kindergarten activities, two settings that have been frequently involved in COVID-19 outbreaks [30–32]. Little attention has also been reserved for other materials commonly used in the food supply chain. In fact, aluminium, tetrapak, plastic as Polyethylene terephthalate (PET), and glass are employed as food contact materials (FCM) for food packaging. A previous study described the detection of SARS-CoV-2 RNA on the surface of a salmon chopping board [33], albeit no information on the viability of the virus was provided. Furthermore, some authors showed the presence of a viable virus on PET from 72 h up to 96 h [17–19].

So far, some studies have been conducted to assess whether the material properties may influence the viability of SARS-CoV-2 [18,34]. In this context, while some authors observed lower survival of SARS-CoV-2 and other coronaviruses on porous surfaces [17,19,35], another study observed a lack of correlation between material features and virus viability [36]. These contradictious findings highlight the need for further investigations to provide additional information that could be useful to mitigate the possible spread of SARS-CoV-2 through contaminated objects. Thus, the present study aimed to analyze the persistence and stability of SARS-CoV-2 on different porous and non-porous inanimate surfaces of daily use by the assessment of viral load kinetics over time, the viability of the virus, and the residual infectivity of the tested surfaces after artificial contamination. Furthermore, a comparison between two different lineages, B.1 and the BA.1, was carried out to estimate how the emergence of a variant considered as a VOC, could influence the abovementioned virus characteristics.

Several intrinsic and extrinsic factors, both of the virus and of the surface, have been identified to be able to impact virus persistence and survival, such as droplet size, environmental conditions, such as humidity, temperature and UV exposure, as well as virus species and strain [19,34,35]. In particular, it has emerged that the persistence of SARS-CoV-2 may be influenced by intrinsic chemical–physical material properties [18,34]. In effect, our results demonstrate a different viral behaviour among the tested materials, related both to the porosity of the surfaces and the lineage used to perform the artificial contamination. Thereby, a difference in persistence and stability between porous and non-porous materials was clearly distinguishable, corroborating the thesis that porosity plays a key role in the dynamic of SARS-CoV-2 on the surfaces [34,37]. A main property of a non-porous surface can be attributed to the limited dispersion of the medium of the droplet in which the virus is suspended, becoming responsible for preserved virus stability [34,37]. In particular, on EVA, EPS, aluminium, PET, glass, and plexiglass smooth materials, by the association of the development of CPE in cell monolayers and the increase in viral loads between the surfaces and cell cultures, it was possible to ascertain a prolonged virus stability and viability. Furthermore, Mautner and colleagues (2022) describe that the visual appearance of CPE in Vero E6 cells correlates well with SARS-CoV-2 maximum infectivity [38]. Thereby, our observations could be related to a preserved infectivity of a live and viable virus. Following this statement, we observed that the most suitable surface that sustained virus stability and preserved virus viability was Ethylene Vinyl Acetate, where SARS-CoV-2 was able to survive up to 72 h post-artificial contamination.

By contrast, the survival of both lineages was rapidly impaired on cotton and polyester. These porous materials, by reabsorbing the medium of the droplet, may have led to a rapid deterioration of the microenvironment [34], making the virus itself less stable. This aspect was extremely evident in polyester and cotton, two materials frequently used in the textile industry, which showed no detectable RNA as soon as 24 and 48 h, respectively, and the virus was not able to survive as soon as 30 min post-artificial contamination. Thus, no CPE developed on cells and the recovered RNA on the surfaces was markedly decreased compared to other non-porous surfaces. Thereby, a very low risk for indirect infection can be attributed to such materials, as already described by Virtanen and colleagues [39]. Cardboard also rapidly impaired SARS-CoV-2 stability, as already observed by van Doremalen and colleagues [17]. Indeed, only the inoculum obtained 30 min after artificial contamination was able to induce CPE in cell monolayer, while as soon as 24 h, either the development of CPE or the increase in viral load was not revealed. The scarce viability was more markedly visible for the omicron variant, which showed a sharp reduction of the viral load just at T1.

Interestingly, in all the tested materials, the cythopathic effect developed by the lineage BA.1 was less intense than that of the B.1, corroborating the data obtained both *in vitro* and *in vivo,* which report lower pathogenicity of the omicron variant than previous circulating lineages, demonstrating highly decreased fusogenicity and syncytia formation [3,4].

Nevertheless, although the use of laboratory features to mimic as much as possible real-life conditions, the use of a dose of 50 μL of 10^5^ TCID50/mL for artificial contamination has been the equivalent titre of the upper respiratory tract of infected individuals [40,41], as well as typically indoor temperature and relative humidity parameters, the results cannot be interpreted as a statement of risk of exposure with fomites but these results can be a reference for the management of closed and densely populated settings and materials used in everyday life. Indeed, lower risks in the real living environment may be possible [42], which some authors considered very small and limited only in particular circumstances where an infected person coughs or sneezes directly on a surface and soon after, within 1-2 h, someone else touches that surface [43]. Applying this concept to crowded settings, such as supermarkets and wholesale markets, where an infected person could uncontrollably sneeze or cough on food products and packaging [44], according to our results the risk connected with these FCMs may extend up to 48 h. Furthermore, higher risk could be represented by children’s behaviour, due to their habit of frequently placing objects into their mouths, which might make infants especially exposed to viral infection [45] in kindergarten. Moreover, in offices and shops, plexiglass barriers have been extensively used to avoid direct contact with droplets. A previous study showed that these barriers without openings can reduce exposure to aerosolized substances [46]. Our study indicates that SARS-CoV-2 remains viable on this material for 48 h and since plexiglass barriers usually contain slots, the risk of exposure is not null [46]. All these aspects have led the European Centre for Disease Prevention and Control (ECDC) to recommend hygiene and disinfection measures for frequently touched surfaces, thereby suggesting the need for continuous disinfection procedures to decrease the risk of contagion through fomites [7]. Nevertheless, the resolution of the mandatory use of masks in close settings might represent a critical aspect for the spread of the virus through fomites.

## Conclusion

Several authors investigated the potential role of surfaces in the indirect transmission of SARS-CoV-2. Considering our findings, particularly, materials (fomites) to date are not yet fully explored, it is conceivable that repeated and promiscuous exposure to these infected materials, such as in kindergarten, gyms, and markets, may contribute to the spread and maintain prolonged SARS-CoV-2 outbreaks. Moreover, higher attention should be addressed towards indirect contact, as the current circulation of the omicron variant shows higher transmissibility, persistence, and viability on surfaces of several commonly used materials, which could represent a potential source of infection and spread of the virus.

## References

[1] Guo Y, Han J, Zhang Y, et al. SARS-CoV-2 omicron variant: epidemiological features, biological characteristics, and clinical significance. Front Immunol. 2022;13:877101. doi:10.3389/fimmu.2022.877101 35572518 PMC9099228

[2] Cameroni E, Bowen JE, Rosen LE, et al. Broadly neutralizing antibodies overcome SARS-CoV-2 Omicron antigenic shift. Nature. 2022;602(7898):664–670. doi:10.1038/s41586-021-04386-2 35016195 PMC9531318

[3] Fan Y, Li X, Zhang L, et al. SARS-CoV-2 Omicron variant: recent progress and future perspectives. Signal Transduct Target Ther. 2022;7(1):141. doi:10.1038/s41392-022-00997-x 35484110 PMC9047469

[4] Bálint G, Vörös-Horváth B, Széchenyi A. Omicron: increased transmissibility and decreased pathogenicity. Signal Transduct Target Ther. 2022;7(1):151. doi:10.1038/s41392-022-01009-8 35525870 PMC9077027

[5] Gupta R. SARS-CoV-2 omicron spike mediated immune escape and tropism shift. Res Square. 2022: rs.3.rs-1191837.

[6] Hirose R, Itoh Y, Ikegaya H, et al. Differences in environmental stability among SARS-CoV-2 variants of concern: both omicron BA.1 and BA.2 have higher stability. Clin Microbiol Infect. 2022;28(11):1486–1491. doi:10.1016/j.cmi.2022.05.020 35640841 PMC9144845

[7] European Centre for Disease Prevention and Control (ECDC). Country overview report: week 33 2022. [cited 2022 Aug 31]. Available from: https://www.ecdc.europa.eu/en/covid-19/country-overviews.

[8] Leung NHL. Transmissibility and transmission of respiratory viruses. Nat Rev Microbiol. 2021;19:528–545. doi:10.1038/s41579-021-00535-6 33753932 PMC7982882

[9] Hu Q, He L, Zhang Y. Community transmission via indirect media-to-person route: a missing link in the rapid spread of COVID-19. Front Public Health. 2021;9:687937. doi:10.3389/fpubh.2021.687937 34395365 PMC8355519

[10] Kutter JS, Spronken MI, Fraaij PL, et al. Transmission routes of respiratory viruses among humans. Curr Opin Virol. 2018;28:142–151. doi:10.1016/j.coviro.2018.01.001 29452994 PMC7102683

[11] Boone SA, Gerba CP. Significance of fomites in the spread of respiratory and enteric viral disease. Appl Environ Microbiol. 2007;73:1687–1696. doi:10.1128/AEM.02051-06 17220247 PMC1828811

[12] Stephens B, Azimi P, Thoemmes MS, et al. Microbial exchange via fomites and implications for human health. Curr Pollution Rep. 2019: 198. doi:10.1007/s40726-019-00123-6 PMC7149182 34171005

[13] Cai J, Sun W, Huang J, et al. Indirect virus transmission in cluster of COVID-19 cases, Wenzhou, People’s Republic of China, 2020. Emerging infect Dis. 2020;26:1343–1345. doi:10.3201/eid2606.200412 PMC7258486 32163030

[14] Galbadage T, Peterson BM, Gunasekera RS. Does COVID-19 spread through droplets alone? Front Public Health. 2020;8:163. doi:10.3389/fpubh.2020.00163 32391310 PMC7193306

[15] Joonaki E, Hassanpouryouzband A, Heldt CL, et al. Surface chemistry can unlock drivers of surface stability of SARSCoV-2 in a variety of environmental conditions. Chem. 2020;6:2135–2146. doi:10.1016/j.chempr.2020.08.001 32838053 PMC7409833

[16] Riddell S, Goldie S, Hill A, et al. The effect of temperature on persistence of SARS-CoV-2 on common surfaces. Virol J. 2020;17(1):145. doi:10.1186/s12985-020-01418-7 33028356 PMC7538848

[17] Van Doremalen N, Bushmaker T, Morris DH, et al. Aerosol and surface stability of SARS-CoV-2 as compared with SARS-CoV-1. N Engl J Med. 2020;382:1564–1567. doi:10.1056/NEJMc2004973 32182409 PMC7121658

[18] Kwon T, Gaudreault NN, Richt JA. Environmental stability of SARS-CoV-2 on different types of surfaces under indoor and seasonal climate conditions. Pathogens. 2021;10(2):227. doi:10.3390/pathogens10020227 33670736 PMC7922895

[19] Chin AWH, Chu JTS, Perera MRA, et al. Stability of SARS-CoV-2 in different environmental conditions. Lancet Microbe. 2020;1(1):e10. doi:10.1016/S2666-5247(20)30003-3 32835322 PMC7214863

[20] Xie C, Zhao H, Li K, et al. The evidence of indirect transmission of SARS-CoV-2 reported in Guangzhou, China. BMC Public Health. 2020;20(1):1202. doi:10.1186/s12889-020-09296-y 32758198 PMC7403788

[21] Guo ZD, Wang ZY, Zhang SF, et al. Aerosol and surface distribution of severe acute respiratory syndrome coronavirus 2 in hospital wards, Wuhan, People’s Republic of China, 2020. Emerg Infect Dis. 2020;26:1583–1591. doi:10.3201/eid2607.200885 32275497 PMC7323510

[22] Mondelli MU, Colaneri M, Seminari EM, et al. Low risk of SARS-CoV-2 transmission by fomites in real-life conditions. Lancet Infect Dis. 2021;21(5):e112. doi:10.1016/S1473-3099(20)30678-2 33007224 PMC7524520

[23] Karber G. Beitrag zur kollecktiven Behandlung pharmakologischer Reihenversuche. Arch Exptl Pathol Pharmakol. 1931;162:480–483. doi:10.1007/BF01863914

[24] Heilingloh CS, Aufderhorst UW, Schipper L, et al. Susceptibility of SARS-CoV-2 to UV irradiation. Am J Infect Contr. 2020;48(10):1273–1275. doi:10.1016/j.ajic.2020.07.031 PMC7402275 32763344

[25] Bullen CK, Davis SL, Looney MM. Quantification of infectious SARS-CoV-2 by the 50% tissue culture infectious dose endpoint dilution assay. Methods Mol Biol. 2022;2452:131–146. doi:10.1007/978-1-0716-2111-0_9 35554905

[26] Cardillo L, de Martinis C, Viscardi M, et al. SARS-CoV-2 quantitative real time PCR and viral loads analysis among asymptomatic and symptomatic patients: an observational study on an outbreak in two nursing facilities in Campania Region (Southern Italy). Infect Agent Cancer. 2021;16(1):45. doi:10.1186/s13027-021-00388-x 34158108 PMC8218569

[27] Onakpoya IJ, Heneghan CJ, Spencer EA, et al. SARS-CoV-2 and the role of fomite transmission: a systematic review. F1000 Res. 2021;10:233. doi:10.12688/f1000research.51590.3 PMC8176266 34136133

[28] Ong S, Tan YK, Chia PY, et al. Air, surface environmental, and personal protective equipment contamination by severe acute respiratory syndrome coronavirus 2 (SARS-CoV-2) from a symptomatic patient. JAMA. 2020;323(16):1610–1612. doi:10.1001/jama.2020.3227 32129805 PMC7057172

[29] Jiang XL, Zhang XL, Zhao XN, et al. Transmission potential of asymptomatic and paucisymptomatic severe acute respiratory syndrome coronavirus 2 infections: a 3-family cluster study in China. J Infect Dis. 2020;221:1948–1952. doi:10.1093/infdis/jiaa206 32319519 PMC7188140

[30] Bark D, Dhillon N, St-Jean M, et al. SARS-CoV-2 transmission in kindergarten to grade 12 schools in the Vancouver Coastal Health region: a descriptive epidemiologic study. CMAJ Open. 2021;9(3):E810–E817. doi:10.9778/cmajo.20210106 PMC8432285 34429325

[31] Kim EY, Choe YJ, Park H, et al. Community transmission of SARS-CoV-2 omicron variant, South Korea, 2021. Emerg Infect Dis. 2022;28(4):898–900. doi:10.3201/eid2804.220006 35171760 PMC8962914

[32] Li H, Shankar SN, Witanachchi CT, et al. Environmental surveillance and transmission risk assessments for SARS-CoV-2 in a fitness center. Aerosol Air Qual Res. 2021;21(11):210106. doi:10.4209/aaqr.210106 35047025 PMC8765736

[33] Hayashi T, Aboko K, Mandan M, et al. Molecular analysis of binding region of an ACE2 as a receptor for SARS-CoV-2 between humans and mammals. bioRxiv. 2020, Preprint: 196378. 10.1080/01652176.2020.1823522 PMC7580767 32921279

[34] Guo L, Yang Z, Guo L, et al. Study on the decay characteristics and transmission risk of respiratory viruses on the surface of objects. Environ Res. 2021;194:110716. doi:10.1016/j.envres.2021.110716 33421429 PMC7834477

[35] Aboubakr HA, Sharafeldin TA, Goyal SM. Stability of SARS-CoV-2 and other coronaviruses in the environment and on common touch surfaces and the influence of climatic conditions: a review. Transbond Emerg Dis. 2021;68(2):296–312. doi::10.1111/tbed.13707 PMC7361302 32603505

[36] Biryukov J, Boydston JA, Dunning RA, et al. Increasing temperature and relative humidity accelerates inactivation of SARS-CoV-2 on surfaces. mSphere. 2020;5(4):e00441–20. doi:10.1128/mSphere.00441-20 32611701 PMC7333574

[37] Chatterjee S, Murallidharan JS, Agrawal A, et al. A review on coronavirus survival on impermeable and porous surfaces. Sādhanā. 2022;47(1):5. doi:10.1007/s12046-021-01772-4

[38] Mautner L, Hoyos M, Dangel A, et al. Replication kinetics and infectivity of SARS-CoV-2 variants of concern in common cell culture models. Virol J. 2022;19(1):76. doi:10.1186/s12985-022-01802-5 35473640 PMC9038516

[39] Virtanen J, Aaltonen K, Kivistö I, et al. Survival of SARS-CoV-2 on clothing materials. Adv Virol. 2021;2021:6623409. doi:10.1155/2021/6623409 33927762 PMC8049815

[40] Karimzadeh S, Bhopal R, Nguyen Tien H. Review of infective dose, routes of transmission and outcome of COVID-19 caused by the SARS-COV-2: comparison with other respiratory viruses. Epidemiol Infect. 2021;149:e96. doi:10.1017/S0950268821001084 33849679 PMC8082124

[41] Xue X, Ball JK, Alexander C, et al. All surfaces are not equal in contact transmission of SARS-CoV-2. Matter. 2020;3(5):1433–1441. doi:10.1016/j.matt.2020.10.006 33043292 PMC7538118

[42] Mohamadi M, Babington-Ashaye A, Lefort A, et al. Risks of infection with SARS-CoV-2 due to contaminated surfaces: a scoping review. Int J Environ Res Public Health. 2021;18(21):11019. doi:10.3390/ijerph182111019 34769538 PMC8583529

[43] Goldman E. Exaggerated risk of transmission of COVID-19 by fomites. Lancet Infect Dis 2020;20:892–893. doi:10.1016/S1473-3099(20)30561-2 32628907 PMC7333993

[44] Lu LC, Quintela I, Lin CH, et al. A review of epidemic investigation on cold-chain food-mediated SARS-CoV-2 transmission and food safety consideration during COVID-19 pandemic. J Food Saf. 2021;41(6):e12932. doi:10.1111/jfs.12932 34898751 PMC8646261

[45] Barker J, Stevens D, Bloomfield SF. Spread and prevention of some common viral infections in community facilities and domestic homes. J Appl Microbiol. 2001;91(1):7–21. doi:10.1046/j.1365-2672.2001.01364.x 11442709 PMC7166786

[46] Cadnum J, Jencson A, Donskey C. Do plexiglass barriers reduce the risk for transmission of severe acute respiratory syndrome coronavirus 2 (SARS-CoV-2)? Infect Contr Hosp Epidemiol. 2021;44(6):1010–1013. doi:10.1017/ice.2021.383 34726150

